# Cycle Flux Algebra for Ion and Water Flux through the KcsA Channel Single-File Pore Links Microscopic Trajectories and Macroscopic Observables

**DOI:** 10.1371/journal.pone.0016578

**Published:** 2011-01-31

**Authors:** Shigetoshi Oiki, Masayuki Iwamoto, Takashi Sumikama

**Affiliations:** Department of Molecular Physiology and Biophysics, University of Fukui Faculty of Medical Sciences, Fukui, Japan; Science and Technology Facilities Council, United Kingdom

## Abstract

In narrow pore ion channels, ions and water molecules diffuse in a single-file manner and cannot pass each other. Under such constraints, ion and water fluxes are coupled, leading to experimentally observable phenomena such as the streaming potential. Analysis of this coupled flux would provide unprecedented insights into the mechanism of permeation. In this study, ion and water permeation through the KcsA potassium channel was the focus, for which an eight-state discrete-state Markov model has been proposed based on the crystal structure, exhibiting four ion-binding sites. Random transitions on the model lead to the generation of the net flux. Here we introduced the concept of cycle flux to derive exact solutions of experimental observables from the permeation model. There are multiple cyclic paths on the model, and random transitions complete the cycles. The rate of cycle completion is called the cycle flux. The net flux is generated by a combination of cyclic paths with their own cycle flux. T.L. Hill developed a graphical method of exact solutions for the cycle flux. This method was extended to calculate one-way cycle fluxes of the KcsA channel. By assigning the stoichiometric numbers for ion and water transfer to each cycle, we established a method to calculate the water-ion coupling ratio (*CR*_w-i_) through cycle flux algebra. These calculations predicted that *CR*_w-i_ would increase at low potassium concentrations. One envisions an intuitive picture of permeation as random transitions among cyclic paths, and the relative contributions of the cycle fluxes afford experimental observables.

## Introduction

The ion channel is a molecular device that provides a unique reaction platform for the permeation of ions and water molecules [1]. The channel facilitates ion flux across the membrane while maintaining ion selectivity. This activity is carried out with a rather simple structure named the pore. In an open pore, ions and water molecules diffuse without requiring energy consumption. The crystal structure of the KcsA potassium channel revealed that the pore is narrow, having a diameter close to the potassium ion size [2]. This allows the passage of bare ions and water molecules, but prevents them from passing each other within the pore (single-file permeation) [3]. This dependency of ion and water flux is a fundamental property of the ion channel, and there are several experimental methods for evaluating the coupled flux [3], [4], [5]. The experimental data thus obtained comprises important information on the interaction of ions and water during the permeation process and is useful for investigating the underlying mechanisms of permeation. However, the lack of an adequate theoretical background for the purpose of quantitative examination of the coupling has left the proper interpretation of such valuable data elusive. In this study, we present a theoretical method for evaluating ion and water coupling on a discrete-state diagram using a thermodynamic concept of cycle flux.

During ion permeation, water molecules, both those on permeating ions and those flanked by ions in the narrow pore, are obligatorily transported along with ions. Ion channels simply serve as a geometrical constraint, a single-file pore, through which queues of ions and water molecules stream, leading to coupled flux. The water flux, driven by the difference in the electrochemical potential of ions, is called “electroosmosis” and is an example of free energy transduction, since water is carried uphill [6], [7], [8]. This unique mechanism has been studied thermodynamically. In particular, the streaming potential (*V*_stream_), the reverse phenomenon of the electroosmosis, has been measured electrophysiologically, from which the ratio of the water and ion fluxes during the permeation (the water-ion coupling ratios; *CR*_w-i_) was evaluated [4], [5], [9], [10], [11], [12]. The *CR*_w-i_ value is a signature revealing individual contribution of ion and water molecules in a flowing queue through the pore. These elementary processes cannot be resolved in ionic current measurements, since the high permeation rate makes the observables as the mean behavior of the ion flux. Thus, measurements of *CR*_w-i_ provide unprecedented information on the permeation. However, this data has yet to be related to the underlying permeation mechanisms in quantitative manner.

**Figure 1:**
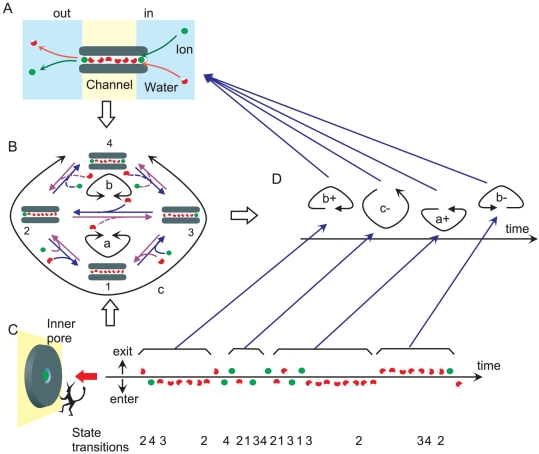
Permeation processes through a single-file pore. **A**. A schematic model for a narrow pore that allows passage of ions and water molecules only in single-file. **B**. A discrete-state Markov model for the channel having two binding sites. The two binding sites are either ion-occupied or not. There are four states, including the double-occupancy state. Tracing transition arrows (violet or blue) leads to cyclic paths (cycle *a*, *b* and *c*), and transitions around cycles generate the net flux (cycle flux). **C**. A Maxwell's demon stays at the inner pore entrance and takes account of the ion and water molecules in and out of the pore during the net influx. A time series of the random elementary steps (entering or exiting of either an ion or a water molecule) noted by the demon is shown. **D**. A time series of cycles deduced from a random sequence of ion and water pop-ups. A piece of the random sequence was lumped together and assigned to one of the one-way cycles, and the random sequence of ion-and-water pop-ups was converted to a series of one-way cycles. Detailed trajectories are reproduce by two demons in both sides of the pore. The cyclic sequence provides a more visual image of permeation processes than the simple random sequence. The sum of cycle fluxes gives the net flux.

To examine *CR*_w-i_ quantitatively, we considered the discrete-state Markov model (DSMM) for ion permeation, on which the concept of the cycle flux was introduced. In earlier studies, electrophysiologists have constructed DSMMs intuitively in an effort to quantitatively account for the experimental single-channel current amplitudes [13], [14], [15], [16]. The position of ion binding site(s) was estimated and the transition paths among the states were proposed. The rate constants were estimated from the experimental data of the current amplitudes. Here, the DSMM for ion permeation is introduced using a microscopic approach (Fig. 1). One way to picture this is to imagine a movie of ions and water undergoing permeation through a single-file pore, such as in the gramicidin channel (Fig. 1A). Computer simulation has revealed all the detailed trajectories of ions and water molecules undergoing permeation [17], [18], [19], [20], [21], [22], [23], [24]. Ions and water molecules in the pore tend to stay in preferred locations, rather than assuming a diffused distribution along the pore. Ensembles of these snapshots reveal that the distribution of ion and water molecules is segregated into a limited number of distribution “states”. The coarse graining of these states and assignment of the transition paths give the transition rates between states. These states and transitions can be represented diagrammatically (Fig. 1B), including the first-order rate constant *k*_ij_ for each possible transition i → j between states. This is a DSMM for ion permeation.

In DSMM, the states on the diagram represent the ion occupancy states in an open pore. In the case of the KcsA channel, the crystal structure revealed that ions are located in four potential binding sites in the selectivity filter (for example PDB code: 1k4d) [25]. This justified the use of the discrete model of ion permeation [26]. The discrete diagram thus constructed serves the purpose of expressing the permeation processes quantitatively.

The concept of the cycle flux was introduced by T.L. Hill [27]. On the state diagram, the net ion flux is represented by a random walk (Fig. 1B). There are multiple cyclic paths on the diagram. For example, an ion enters from the right side (transition 1 → 3), then, the ion moves from the right binding site to the left (3 → 2). The ion exits to the left side (2 →1) and completes a cycle *a*. Each cycle represents how ion and water molecules are transferred. The rate of completing a cycle is called cycle flux [28]. Cycle flux is driven by the free energy conjugated to the cycle [27], [29]. In a complex diagram, there are many possible cyclic paths but not all the cycles generate non-zero net flux. During a random walk, selecting a cycle or cycles from among the others determines the macroscopic observables. Therefore a structure of permeation emerges on the DSMM by introducing the concept of cyclic paths and the cycle flux.

How is the cycle flux evaluated? The stochastic processes of ion permeation jumping from one state to another on DSMM have been simulated with a Monte Carlo method, and counting numbers of cycle completion gave an approximate solution of the cycle flux [26], [28]. On the other hand, Hill developed a diagrammatic method for obtaining the exact solution for the one-way cycle flux [27], [30]. This graphical method has been applied to simple diagrams, but has not been applied to more complicated diagram such as that for the KcsA potassium channel. This is the challenge taken up by this study.

The concept of the cycle flux is visualized in the following way (Fig. 1C,D). We assume the view point of “Maxwell's demon”, who focuses on the inner entrance of the pore and takes into account the ions and water molecules coming into and going out of the pore (Fig. 1C). During a net outward ionic flux, for example, the demon is concerned with only entering and exiting traffics, rather than with the detailed trajectories towards the pore entrance. The observed queue of ion and water molecules is shown. This queue can be transformed into a cyclic process on the diagram (Fig. 1D): A short stretch of the sequence is assigned to a cycle of one-way directionality. Continuing this assignment leads to the generation of a sequence of cycles. Thus, the whole random sequence of ion-water pop-up animation (Fig. 1C) can be translated into random transitions among cycles (Fig. 1D). The sequence of cycles recapitulates the permeation process, while retaining the essential information on the permeation.

Researchers participating the molecular dynamics for ion permeation studies have more information than the demon has. For potassium channels, huge calculation has been performed, and researchers supported the knock-on mechanism for explaining the efficient permeation [19], [20], [22]. From huge numbers of microscopic trajectories, understanding underlying processes are, however, elusive. Our proposal in this study is to provide a mesoscopic view point for analyzing permeation processes [31].

We start our analysis by applying the diagram method to the gramicidin A channel as a lesson, since the diagram is simple and the analytical solution is available. Then, the cycle flux was calculated for the first time for the KcsA potassium channel. Cycle flux evaluation not only gave the exact solution of the water-ion coupling ratio, but it also provided a more intuitive picture of permeation that the random transitions among the cyclic paths underlie the permeation processes.

## Methods

The underlying theory of the cycle flux and the diagrammatic methos are presented.

### The theory of the cycle flux

The physical processes of ion permeation through the channel in real space can be projected onto a random walk in the discrete-state diagram (Fig. 1). After a long random walk, the time spent in the state *i* gives the steady-state probability (*p*_i_), which can be calculated algebraically from the rate constants of the diagram (matrix inversion) [27]. Under the steady-state condition, the net reaction flux for each transition pair (the transition flux: *k*_ij_ *p*_i_ – *k*_ji_ *p*_j_) is readily calculated. Meanwhile, a random walk on the diagram completes a cycle and the rate of completion is defined as the cycle flux [27]. There are multiple cyclic paths on the diagram, and in the steady state the cycles are completed at their own individual rates. The sum of all the cycle fluxes gives the net flux of the reaction. Thus, a random walk among states on the diagram can be integrated into random transitions among cycle kinetics.

Each cycle κ with the cycle flux of *J*_κ_ is composed of two one-way cycles having directionality for outward flux (*J*_κ+_) and inward flux (*J*_κ–_), where *J*_κ_  =  *J*_κ+_– *J*_κ−_. The cycle flux contains more detailed information than the net flux. The driving force and flux are closely related in a cycle. The ratio of one-way cycle fluxes is given as
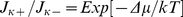
(1)where Δµ represents the electrochemical potential difference for the relevant substances travelling across the membrane [27], [29]. The cycle flux cannot be calculated from *p*_i_ except for simple diagrams, such as that for the gramicidin channel, and a Monte Carlo simulation was needed for the approximate solution. On the other hand, T. L. Hill developed a diagrammatic method for the exact solution of the one-way cycle flux [30].

In general, the elapsed time for completing a cycle has been estimated by applying the mean first passage time. In diagrams having cyclic paths, the objects of interest are random walks that end with the first cycle completion. Hill extracted an essential idea for deriving the mean first passage time and implemented it in a diagrammatic format, named the expanded diagram [30]. Here, the general idea underlying the diagrammatic method is described.

#### The expanded diagram

**Figure 2:**
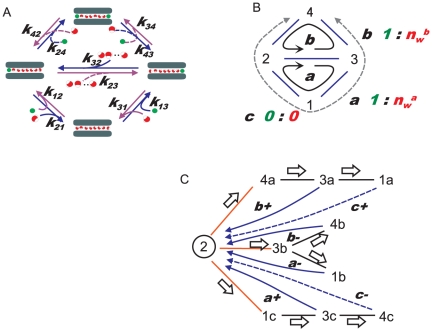
A diagrammatic method for examining ion permeation. **A**. The classical DSMM for the gramicidin channel. The ion occupancy states and the transitions between them are shown with the rate constants. The left side of the channel is assigned as the outside of the membrane, and the right side, the inside. The blue arrows indicate the paths for efflux and the violet ones the paths for influx. Curved lines merging to arrows depict association processes of either ion or water molecules from the inside or outside. For example, the path for the rate constant *k*_13_ associates with an ion from the inside. **B**. Designation of the states (number) and the cyclic paths (alphabet). The arrows indicate the direction of ion efflux, which is defined as the + direction hereafter. The stoichiometric numbers for water (red) and ions (green) for each cycle are shown. Cycle *c* does not give the net flux, since the stoichiometric numbers were zero. **C**. The expanded diagram of the gramicidin model for the one-way cycle flux. State 2 was arbitrarily defined as the starting state. Three routes outflow from state 2 (red), and each is either branched or not. This generates the tree diagram (black solid lines). Then the returning paths to the state upstream, which complete the cycles, are drawn with one-way arrows (blue arrows). Through this procedure all the cycles in the original diagram were decomposed into one-way cycles (*a*+, *a*−, *b*+, *b*−, *c*+ and *c*−), and states (except for the case of 2) were divided into sub-states. Sub-state 1b, for example, is the second sub-state of state 1. The one-way cycle flux for *a*+ can be calculated as *k*_32_ × *P*_3c_.

For a given diagram having multiple cyclic paths (original diagram), of primary concerns are the time-averaged rates of one-way cycle completions. As a simple example, a four-state diagram for the gramicidin A (gA) channel was considered (Fig. 2A). To evaluate the cycle flux, the original diagram was transformed such that each cyclic path was decomposed into two independent one-way cyclic paths (the expanded diagram). Clockwise and counter-clockwise transitions around the cyclic paths on the original diagram were tranformed into two separate independnet paths on the expanded diagram.

First, the starting state *s* was selected. Here we defined the starting state at state 2 (Fig. 2C). Second, independent paths for either directionality of cycles were searched by drawing the tree diagram. The tree diagram was formed by tracing all the states without the formation of cycles (a Hamiltonian path [32]). As shown in Fig. 2C, a transition line out of state 2 for each possible transition option (i.e. to state 1, 3 or 4) is drawn (red lines), and this branching process continues state by state, with a proliferation of paths (Fig. 2C, black lines). A state on the original diagram appeared repeatedly on different paths, which were distinguished from one another by adding a second index (alphabetical) after the original state designation (sub-states). For example, a sub-state of state 3 is observed in one path and designated “3a” and in another path designated “3b”. The sequences of sub-states specify the one-way paths.

Third, an expanded diagram was formed by introducing the returning paths. We are concerned only with time averages over a very long continuous walk. Our object, therefore, is to transform the tree diagram so as to permit a continuous walk on a new diagram that duplicates the state transition choices in the continuous walk on the original diagram. This is accomplished by returning each cycle-completion arrow to the state or sub-state that originated the cycle just completed (Fig. 2C, blue arrows). These procedures eventually generate a new diagram (expanded diagram), with all the cyclic paths having a single direction (one-way cycles). The expanded diagram is thus a more-detailed version of the original diagram. The cycles of the original diagram are subdivided into one-way cycles. The transition paths and transition rates are the same at every step for a walk on the original and expanded diagram. That is, the two walks are essentially identical. Hence, each cycle type is completed at the same rate on the two diagrams.

#### One-way cycle flux

For a given cycle, the number of cycle completion, or the cycle flux, can be counted when random transitions pass through the one-way return path. The cycle flux is formalized as the mean first passage time [30]. Let *P*_i_(*t*) be the fraction of walks that are in state *i* at *t* on the expanded diagram. In principle, all the *P*_i_(t) can be found by solving the set of linear first-order differential equations, with constant coefficients, of the form
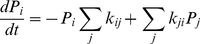
(2)where *k*_ij_ is the rate constant for the transition from the *i* state to the *j* state, and the sums here are over those states *j* that can convert to state *i* upon a transition.

For the mean first passage time or first cycle completion, absorbing states are introduced. The absorption states are assigned as the states at the end of the one-way return arrows in the expanded diagram. The one way return paths in the expanded diagram secures the repeated trials of random walks for completing the cycles on a single diagram over a very long time. In the tree diagram, on the other hand, a large ensemble of walks all starting at *t*  = 0 is assumed to obtain ensemble averages. Thus, the expanded diagram is based on the time-averaging method. The fraction of the walks that ends at absorbing state *m* between *t* an *t* + *dt* is
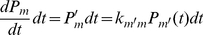
(3)where *k*_m'm_ is the transition rate from *m*' to *m*, where *m*' is the immediate precursor of the absorption state *m*. Here *m*s represent the states at the end of the one-way return arrows. For example, state 2 is the absorbing state (Fig. 2C), but multiple absorbing states exist in more complicated models. In this model, *m*' is state 3a and *m* is state 2 for cycle *b*+ (Fig. 2C). Equation 3 is applied to the absorbing paths (blue arrows in Fig. 2C). In the expanded diagram, *P*_i_ (the steady-state probability of the sub-state *i*) is the fraction of time spent in state *i* in the long repeated random walk. *P*_i_s are obtained from the inversion of the matrix for the expanded diagram. Having found the *P*_i_ for the diagram, the one-way cycle flux, *J*_κ±_, is obtained from *k*_m'm_ *P*_m'_. In general, there are multiple cyclic paths in the expanded diagram for a given cycle of the original diagram. The mean rate of κ cycle completions is *J*_κ±_  =  Σ_κ±_ *k*_m'm_ *P*_m'_.

In summary, the systematic method of drawing the expanded diagram enabled tracing of the independent paths for cycles of both clockwise and counter-clockwise directionality, and the states in the original diagram were decomposed into the sub-states. The path allocated the steady-state probability of the sub-states, and these path-dependent sub-states define the cycle flux.

#### Stoichiometric numbers and non-zero cycle fluxes

To calculate the net flux, stoichiometric numbers for each cycle are prerequisite. There are three cyclic paths for the original gA diagram (Fig. 2B), which were decomposed into six one-way cyclic paths in the expanded diagram (Fig. 2C). For each cycle, the net number of ion and water molecules carried around a cycle (*n*_i_ and *n*_w_) were tallied (Fig. 2B). For cycle *a* and *b*, the *n*_i_ value was the same, while the *n*_w_ value was set differently.

### The diagrammatic methods

The cycle flux was calculated with the following procedure. Step 1: The expanded diagrams were drawn, on which the names of the one-way cycles are assigned. All the cyclic paths were identified through this systematic procedure of the diagram drawing. Step 2: The transition matrix was formulated based on the expanded diagram, and the matrix inversion was performed for the steady-state probability of the sub-states. Step 3: The stoichiometric numbers for the ion and water molecules were counted for each cycle. Step 4: The one-way cycle fluxes were calculated as follows. For each one-way cyclic path, the completing transition indicated by an returning arrow was focused. The probability of the sub-state on the base point of the arrow was multiplied by the transition rate indicated by the arrow direction, which gave the cycle flux. For one-way cycles having multiple paths, the cycle flux was calculated as the sum of the cycle flux for the paths.

#### The gramicidin A channel

In the expanded diagram (Fig. 2C), there are all together ten sub-states including state 2 (three sub-states for state 1, 3 and 4), and the transition matrix having 10×10 dimensions was formulated (Eq. 4). Each element of the matrix was filled with the rate constant for relevant transition between sub-states based on the expanded diagram. For example, the first element of the fifth column is filled with the rate constant from *S*_1a_ to *S*_3a_, i.e., *k*_13_. This rate constant is the same with transitions from *S*_1_ to *S*_3_ in the original diagram. The same rate constant was used for transition of *S*_1b_ → *S*_3b_ (the second element of the sixth column) and *S*_1c_ → *S*_3c_ (the third element of the seventh column). The steady state probability of the sub-states was calculated from the matrix inversion either analytically or numerically. The bold elements in the matrix are the rate constants for absorbing transitions.
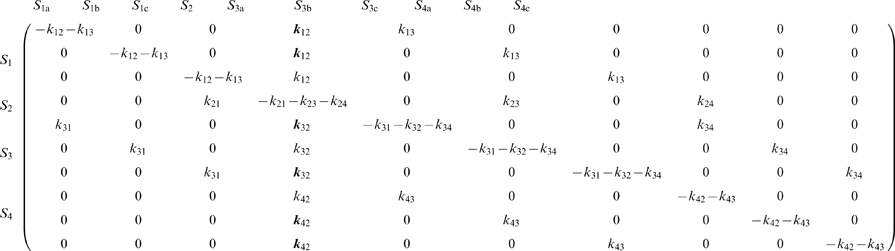
(4)


The transition matrix for the original 4-state diagram is simple,
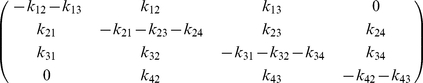
(5)the rate constants for which (Fig. 2A) have been reported under various experimental conditions. Here, the set of rate constants for Rb^+^ permeation was used, since the gA channel exhibits typical multi-ion nature for Rb^+^ permeation (Table 1) [33], and significant contribution of cycle *b* was expected. The voltage-dependence was implemented as *k*_ij_(*V*)  =  *k*_ij_^0^ Exp[-*d*_ij_ *e V*/*kT*] (*k*_ij_^0^ is the rate constant at 0 mV, *d*_ij_ is the electrical distance, *e* the elementary charge, *V* the membrane potential, *k* the Boltzmann constant and *T* the absolute temperature). The structure of the gA channel is symmetrical, and the free energy profile should be a mirror image at 0 mV, hence the rate constants for the symmetrical paths are also the same at 0 mV, such as *k*_23_^0^ =  *k*_32_^0^. For transitions associating with either ion or water, the rate constant becomes *k*_ij_(*V*)  =  *c k*_ij_^0^ Exp[-*d*_ij_ *e V*/*kT*], where *c* represents the ion concentration of the bulk solution. A matrix inversion was performed for the steady-state probabilities of sub-states (*P*_i_) both algebraically and numerically using Mathematica (Wolfram Research, Champaign, IL). The algebraic solution for *P*_i_ is given in the Appendix S1. The sum of *P*_i_ from the expanded diagram should be equal to that of the states (*p*_i_) obtained from the original diagram, which was indeed confirmed by the algebraic results.

**Table 1:** The rate constants for the permeation model of the gramicidin channel.

Experimental conditions Rb^+^	*k*_32_ (10^7^ ms^−1^)	*k*_13_ (M^−1^ ms^−1^)	*k*_21_ (ms^−1^)	*k*_24_ (M^−1 ^ms^−1^)	*k*_43_ (M^−1^ ms^−1^)
*k*_i_^0^	4.5×10^7^	9.0×10^7^	0.9×10^7^	9.0×10^7^	9.9×10^7^
*Electrical distance*	0.39	0.04	0.07	0.04	0.07

The rate constants at 0 mV (*k*_ij_^0^) and their voltage dependency, as indicated by the electrical distance, are shown. The electrical distance is a fraction of the membrane potential. The potential energy profile is assumed to be symmetrical, and the rate constants for symmetrical paths are set identical.



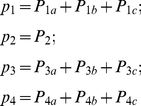
(6)Occupancy probability of ions at each four binding site was calculated from *p*_i_s.

Before calculating the one-way cycle flux, the stoichiometric number was examined (Fig. 2B). It turned out to be zero for cycle *c*, and needs not to be considered for the cycle flux. From the expanded diagram, the one-way cycle flux (*J*_κ±_) can be calculated as the product of a sub-state at the base of a returning arrow and the rate constant for the returning arrow (Fig. 2C).
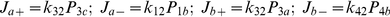
(7)The net flux was calculated from the cycle flux,
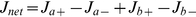
(8)On the other hand, the net flux is also calculated from the original diagram using the transition flux (*J*_ij_) from one state to another, which is defined from the steady-state probability of the states, rather than the sub-states.
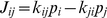
(9)The transition flux is related to the cycle fluxes such that cycles passing through a relevant transition path are summed up [27]. Thus,
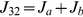
(10)This relation confirmed that the net flux can be calculated from the original diagram in the case of the gA model.

#### The KcsA potassium channel

**Figure 3:**
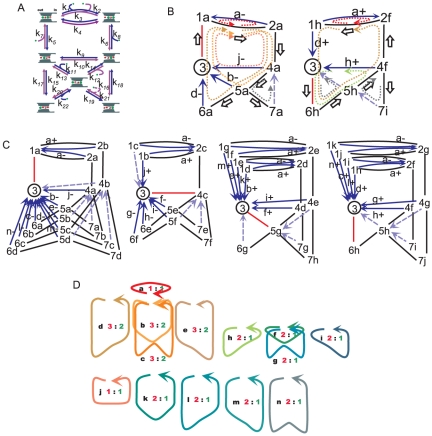
A kinetic model for ion and water permeation through the KcsA potassium channel. **A**. A DSMM for the KcsA channel. The dark blue arrows indicate the transitions for efflux, and the violet for influx. **B**. Two examples of the expanded diagram. For the left diagram, transitions started from state 3 to state 1a (red line), and a trajectory of the branching tree is indicated by the white arrows. At each sub-state, returning paths for completing cycles were found. For each cycle, stoichiometric numbers were calculated. Among them, a cycle 4a → 5a → 7a → 4a gave the stoichiometry of zero for both ion and water flux, and thus it was not named. **C**. The expanded diagram. This diagram was constructed by setting the starting state as 3, from which the red lines were drawn for the initial transitions. The numbers indicate the state number followed by the sub-state alphabetical character. The arrows indicate the last transitions for completing a cycle. The solid arrows indicate the completing transitions for non-zero cycles, and they are labeled with the cycle names. The broken arrows are the completing transitions for cycles generating no net flux. There are six sub-cycles for cycle *a*+ and *a*−, but the rest of the cycles have only one. **D**. The cycles contributing to the non-zero net flux. The arrows indicate the direction of net efflux. The water-ion coupling ratios were assigned for each cycle. The green numbers represent the stoichiometric number for the ions and the red represent the water molecules.

Morais-Cabral et al. proposed the eight-state permeation model for the KcsA channel from the ion distribution in the selectivity filter of several crystal structures [26]. In their model, either an ion or a water molecule occupies one of the four binding sites, and two ions are not allowed to occupy adjacent positions because of electrostatic repulsion. We hereafter call this model the canonical KcsA model. Morais-Cabral et al. subsequently further simplified the model, and the transition paths to which they assigned the rate constants are shown (Fig. 3A). Recently, “atypical” ion distributions not involved in the canonical model were observed in the study of molecular dynamics [24]. To include these states, the canonical model must be expanded, which is an issue of our undergoing study, but it is the outside of the scope of this paper.

Calculating cycle fluxes for the canonical KcsA model is challenging, and it has not been reportedly performed previously. In contrast to the simple gA channel, we addressed two crucial points for the multiply branched KcsA model. First, an encompassing of all the possible routes for drawing the expanded diagram requires careful tracing on the diagram. In the tracing procedure, connectivity between states is the main concern, and the directionality of the transition paths in the original diagram needs not be an issue. Second, among the one-way cyclic paths, some of them generate non-zero cycle flux and others do not, which is identified by the net numbers of transfer counted around the cycles (stoichiometric numbers). The above issues have not been areas of concern in the simpler models [27].

#### The expanded diagram for the KcsA channel

Drawing the expanded diagram was initiated from state 3, which has the largest number of transition paths to other states and, we found that this selection generated the simplest expanded diagrams. The initial transitions out of state 3 for each possible transition option (to state 1, 4, 5 and 6) are colored red (Fig. 3C). Tracing all the states without the formation of cycles (a Hamiltonian path [32]) generated growing numbers of branched paths (a tree diagram). On the tree diagram, returning paths were searched along the branched paths, and they are drawn with one-way arrows (blue arrows). This procedure generates the expanded diagram. On the expanded diagrams, one-way cycles were named on the blue arrows.

Two examples with detailed branching paths (white arrows) are shown in Fig. 3B. In the left diagram, transitions started from state 3 to 1a and traced through 2a, 4a and 5a, where it branched to 7a and 6a. Along this tree diagram, returning arrows were drawn at each state. One-way cycles, cycle *a*–, *j*–, *b*–, *d*–, were formed. Similarly, in the right diagram an initial transition towards state 6h leads to generation of cycles having + directionality.

Altogether, each state was divided into as many as eleven sub-states: 11 sub-states for state 1; 10 for state 7; 8 for state 5 and 6; 7 for state 2 and 4; and 1 for state 3 (Fig. 3C). There were 52 sub-states. The rate constants for transitions between the sub-states were filled on the matrix of 52×52 dimensions according to the expanded diagram.

#### Non-zero cycles

All the possible cycles on the original diagram were identified on the expanded diagram (Fig. 3C). To find cycles exhibiting non-zero cycle flux (non-zero cycles), the following procedure was taken. Some non-zero cycles were identified upon visual inspection as one-way cycles formed with arrows of the same color around a cyclic path on the original diagram (either blue or violet in Fig. 3A). These cycles definetely generate non-zero cycle flux. For example, cycle *f* of either of the one-way directions is formed by three arrows of the same color. There are nine cyclic paths satisfying this criteria (from *a* to *i* cycles) (Fig. 3D upper row) [11].

To determine other non-zero cycles, the net number of ion and water molecules transferred in a cycle was counted. Among cycles returning to state 3, there are cyclic paths involving transition arrows of blue and violet colors, while they produce non-zero cycle fluxes. Those cycles are shown in the lower portion of Fig. 3D. The rest of the cycles (examples are shown in Fig. 3B with the black dotted arrows) did not generate the cycle flux. The examination was successfully completed (Fig. 3 C). Altogether there are 14 non-zero cycles. For each cycle, a stoichiometric number (water : ion) was assigned.

#### The steady-state probability of sub-states and fluxes

The matrix inversion was performed numerically using Mathematica and all the *P*_i_ values were obtained. For cycle flux calculation, a labeled arrow in the expanded diagram was focused, and the *P*_i_ at the base of the arrow was multiplied by the rate constant for transition indicated by the arrow. For example, cycle *b*– was named on the expanded diagram of upper left (Fig. 3B), where transition started from state 3 to 1a → 2a → 4a → 5a and returned to state 3. The cycle flux of *b*– (*J*_b–_) was calculated from the completing arrow (5a → 3) as *k*_13_ × *P*_5a_. Similarly,
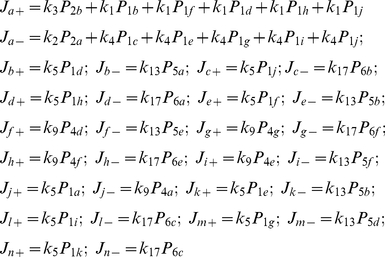
(11)The net flux is defined as the sum of the cycle fluxes.
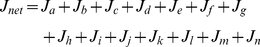
(12)


**Figure 4:**
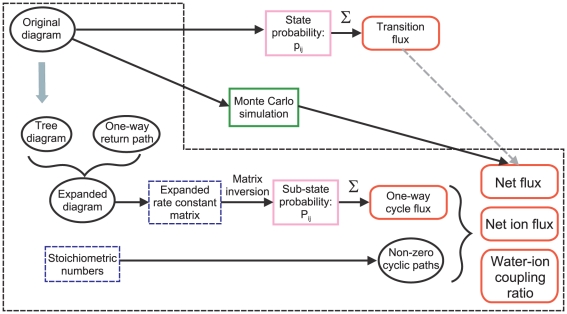
The scheme for the procedure for obtaining the one-way cycle flux. Diagrammatic procedures were indicated by the ovals. Numerical values were indicated by blue boxes. The state and sub-state probabilities were obtained from matrix inversion. The results were indicated by the rounded boxes. The one-way cycle flux and the stoichiometric number gave macroscopic observables, such as the net flux, the net ion flux and the water-ion coupling ratio. Generally, the net flux cannot be calculated from the transition flux, and Monte Carlo simulation provides approximate solutions.

Fig. 4 summarizes the procedure of the cycle flux calculation.

## Results

### One-way cycle flux in the gramicidin channel

In this study, the classical four-state DSMM for the gA channel was applied and the water permeation was implemented into the model by introducing the stoichiometric number (*n*_w_) (Fig. 2A and B). The general solution for this simple diagram has been obtained previously [13], [15], [33]. Here, the results for gA channel using the cycle flux calculation are demonstrated. Among the cycles, the *n*_i_ value is one for cycle *a* and *b* and zero for cycle *c*. Thus, two cycles (*a* and *b*) contribute to the net ion flux (Fig. 1B), while cycle *c* gives zero-flux.

**Figure 5:**
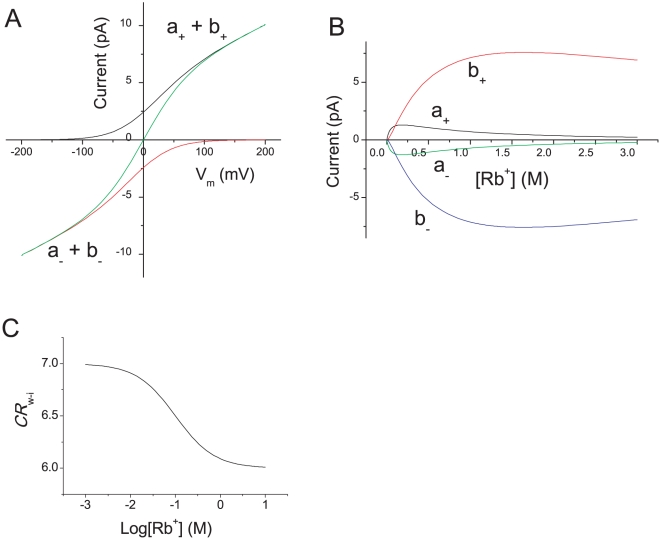
Calculated permeation characteristics of the gramicidin model. **A**. The current-voltage curves at the Rb^+^ concentration of 1 M. **B**. All of the one-way cycle fluxes are a function of the Rb^+^ concentration at 0 mV. **C**. *CR*_w-i_ is a function of the Rb^+^ concentration.

The net ion flux was calculated from either the original or the expanded diagram using the same set of rate constants used for Rb^+^ permeation, for which the gA channel exhibits a multi-ionic character. As shown in Fig. 5A, the net flux as a function of the membrane potential obtained from the original diagram and the expanded diagram turned out to be identical (green). This is shown algebraically from the solution of the cycle flux (see Appendix S1).

One-way cycle fluxes of plus (black) and minus (red) directions were calculated from the expanded diagram, which are a decomposition of the net ion flux (Fig. 5A). In Fig. 5B, each component of the one-way cycle flux at different Rb^+^ concentrations is shown. As the Rb^+^ concentration increased, cycle *b* came to predominate.

There are either six or seven water molecules within a gramicidin channel [4], [5]. Here, different stoichiometry for water and ion transfer (*n*_w-i_) was assumed for each cycle (Fig. 1B). We assume that the left ion in the state 2 is exchanged by a water molecule from the left bulk (transition 2 → 1) and a water column is pushed when an ion in the right bulk binds to the right side (transition 1 → 3). Then the number of water molecules transferred in cycle *a* (*n*_w_^a^) is one more than that for cycle *b* (*n*_w_^b^). The *n*_w-i_^b^ was set to six, while *n*_w-i_^a^ was seven. The water-ion coupling ratio (*CR*_w-i_) was calculated as a function of the Rb^+^ concentration. It decreased gradually, which is consistent with the earlier experimental reports [4], [5].

### Flux calculation for the KcsA potassium channel from the original diagram

Morais-Cabral *et al.* obtained a set of optimized rate constants for the original diagram (Fig. 3A) from experimental data, including the single-channel current amplitudes and ion distributions in the crystal structure at different potassium concentrations [26]. We followed the assumption posed by Morais-Cabral et al. that the potential profile of permeation was symmetrical, which rendered the number of the free parameters less. Their parameters were used for the following calculations (Table 2). For the original diagram of KcsA channel, the steady-state probability of the states was calculated from the 7×7 matrix (see Appendices) by matrix inversion.

**Table 2:** The rate constants and their voltage dependency for potassium permeation of the KcsA channel.

	*k*_1_	*k*_3_	*k*_5_	*k*_9_	*k*_11_	*k*_13_	*k*_15_	*k*_17_	*k*_19_
*k*_i_^0^	4.0×10^9^	2.0×10^9^	5.0×10^7^	1.0×10^9^	4.0×10^10^	5.0×10^8^	1.0×10^7^	1.0×10^6^	2.5×10^8^
*Electrical distance*	0.3	0.2	0.2	0.1	0.2	−0.2	0.1	−0.1	0.1

The rate constants from Morais-Cabral *et al*. The potential energy profile is assumed to be symmetrical and the rate constants are identical for the symmetrical transition paths.

In contrast to the readily calculated net flux for the gA channel, calculation of the net flux for KcsA from the original diagram needs additional consideration. Algebraically the net flux is simply defined as in Eq. 12. However, without the evaluation of the cycle flux that was the case in the earlier studies, only the transition flux (Eq. 9) was calculated. How is it possible to obtain equivalent results of Eq. 12 from the steady state probabilities of the original diagram? In other words, which transition fluxes should be used for the net flux calculation?

A transition flux, *J*_43_, for example, gives
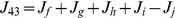
(13)To cover all of the cycle fluxes of Eq. 12 for calculating the net flux, all of the transition fluxes were examined. Among them, the transition paths for *J*_21_^L^ and *J*_12_^U^ (U represents the upper route of cycle *a* using transition paths of *k*_1_ and *k*_2_, while L represents the lower path.) involve relevant cycles.
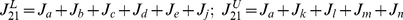
(14)Summing up the above three transition fluxes (*J*_43_+*J*_21_^L^+*J*_21_^U^) gives,
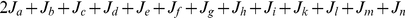
(15)and, unfortunately, this is not equal to Eq. 12. Examining the other linear combinations of the transition fluxes revealed that *J*_net_ could not be attained as a combination of transition fluxes of the original diagram. Absence of the solution was proved exclusively by matrix algebra (Appendix S1).

This procedure taken in this section provides a general rule as to whether the net flux can be calculated from the original diagram or not.

### One-way cycle flux for the KcsA potassium channel

From the expanded diagram of the KcsA channel having a total number of 52 sub-states (Fig. 3C), the rate constant matrix was formulated (Methods). Given the rate constants, the matrix inversion was performed numerically, which did not cost much because of the sparse matrix.

**Figure 6:**
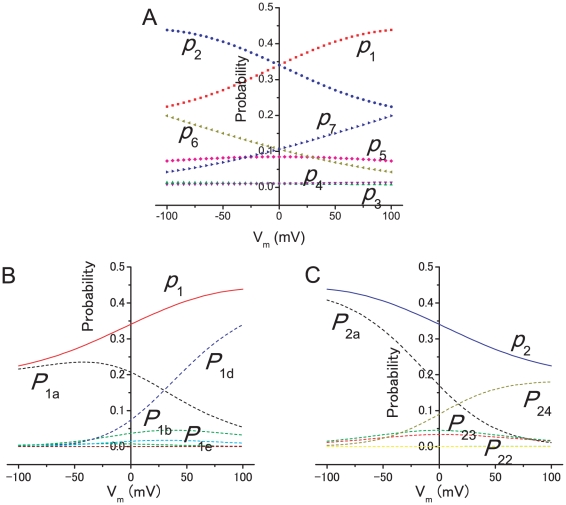
Probability of the states and sub-states for the KcsA channel. **A**. Probability of states at different membrane potentials. The solid lines indicate the probability obtained from the original diagram and the symbols represent the probability calculated from the expanded diagram. **B** and **C**. Probability of state 1 (*p*_1_ in B) and 2 (*p*_2_ in C) and the sub-states. The number of sub-states is 11 for *p*_1_ (*P*_1a_ to *P*_1k_) and 7 for *p*_2_ (*P*_2a_ to *P*_2g_). Only sub-states significantly contributing to the state probability are labeled.

To confirm the validity of the calculation, the steady-state probability of each state was calculated from those of the sub-states (e.g., Eq. 4 for the gA model), and the values were compared with the probabilities calculated from the original diagram. The calculation was performed in an electrolyte solution composed of 0.1 M KCl on both sides of the membrane. In Fig. 6A, the probability of the states is shown as a function of the membrane potential, in which the dotted line demonstrates the results from the expanded diagram, while the solid line is taken from the original diagram. Since the potential profile was assumed to be symmetrical, the curves for the symmetrical states (state 1 vs. 2; 3 vs. 4; 6 vs. 7) are mirror images of one another. In Fig. 6B and C, the probability of the sub-states for states 1 and 2 are shown. The probability of states, *p*_1_ and *p*_2_, were subdivided into different numbers of sub-states. The asymmetric decomposition of *p*_1_ and *p*_2_ into the sub-states seems to be a result of setting the starting state at state 3. These results suggest that the sub-states for the one-way cycle fluxes were successfully calculated from the expanded diagram.

To calculate the macroscopic features, the stoichiometric numbers are crucially important. By examining the permeation processes step by step in the course of each cyclic path, the number of ions and water molecules carried upon completing a cycle was obtained (Fig. 3D).

### The net ionic current

In the previous section, we proved that the exact solution of the net flux cannot be calculated from the original diagrams in the level of complexity for the KcsA diagram. This issue has not been clearly addressed previously since, in the case of the simple diagram such as that for the gA channel, the net flux was readily calculated from the transition flux (Eq. 9). Furthermore, the net ionic current was calculated from the net flux by simply multiplying *z n*_i_ *F*.
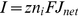
(16)where *z* is the valence, *F* is the Faraday constant, since both cycles *a* and *b* carry a single charge.

The net ionic current for the KcsA diagram is given as the sum of charges carried by cycles, having different stoichiometric numbers (*n*_i_). Thus,
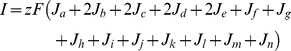
(17)


**Figure 7:**
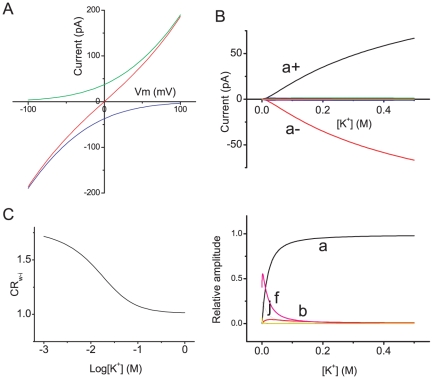
Calculated permeation characteristics of the KcsA channel. **A**. The calculated current-voltage curves for the KcsA channel in the symmetrical 200 mM K^+^ solutions. The + and − one-way cycle fluxes (green and blue) and the net ionic current (red). **B**. Each one-way cycle flux as a function of the K^+^ concentration at 0 mV. Cycles *a*± predominated, and the rest of the cycles were close to zero. Lower: The relative contribution of each cycle. **C**. *CR*_w-i_ as a function of the K^+^ concentration.

Fig. 7 shows the main results of this study. The net ionic current, as well as ionic current by ±one-way cycle fluxes, as a function of the voltage is shown (Fig. 7A). The relative contribution of the cycle fluxes at different potassium concentrations demonstrates that cycle *a* predominated throughout the concentration range (Fig. 7B). Only cycle *f* made a significant contribution at low K^+^ concentrations (Fig. 7B lower). At 3 mM K^+^, the relative contribution of cycle *f* was 0.55, and the probability of state 5 (*p*_5_), in which two ions occupied both ends, was 0.078. The occupancy probability of ions on the four binding sites was 0.462 for both ends and 0.073 for the inner two sites.

### The water-ion coupling ratio

Similar to the net ionic current, *n*_w-i_, the stoichiometric number of water and ion flux, was assigned for each cycle flux (Fig. 3D), from which *CR*_w-i_ was calculated (Fig. 7C). These relationships are expressed in the following way.
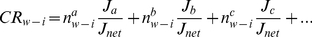
(18)
*CR*_w-i_ is the weighted sum of *n*_w-i_ for each cycle. As the potassium concentration was increased, the *CR*_w-i_ decreased significantly, since cycles having smaller *n*_w-i_ became predominant. This is qualitatively compatible to our previous results for the *CR*_w-i_ values of the HERG potassium channel, that may share this permeation diagram [11]. We predict that the *CR*_w-i_ values of the KcsA channel should increase significantly at low K^+^ concentrations.

The results of the net ionic current and *CR*_w-i_ have been calculated from a particular set of the rate constants, and the validity of the parameter set must be further examined experimentally.

### Cycle flux algebra

**Figure 8:**
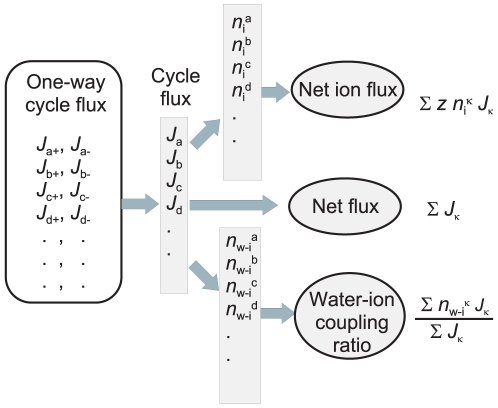
The one-way cycle flux and the macroscopic observables. Assigning the carried charge (*z*_i_) and the stoichiometiric numbers of water and ion (*n*_w-i_) to cycles, the net current and the coupling ratio are readily calculated from the one-way cycle fluxes.

Here we show a general scheme for calculating the net ionic current and *CR*_w-i_ by using one-way cycle fluxes (Fig. 8). The essence of the diagrammatic method for cycle flux is the capacity to decompose the whole permeation diagram into several independent one-way cycles, in which the driving force and the cycle flux are conjugated (Eq. 1). In addition, assigned stoichiometric numbers, such as *n*_i_ and *n*_i-w_, characterize the macroscopic observables. We refer this approach the cycle flux algebra.

## Discussion

For studying ion permeation through the channel, multimodal results for the KcsA channel, such as the crystal structure, the ion distribution in the pore, and the microscopic ion trajectories, have been accumulated. Accompanying with the single-channel current data, our understandings on the ion permeation have advanced dramatically through examining the KcsA channel. However, ion permeation processes cover a broad spectrum of events ranging from the microscopic trajectories of local ion movement to the net ion flux [31], while the available data are far apart in the spatial and temporal regime. Therefore, one cannot integrate those data for drawing full picture of permeation. Here we proposed a novel approach filling in the gaps by introducing the concept of the cycle flux on the DSMM.

DSMM plays an essential role in the integration of information from different levels of the permeation events [31]. On DSMM, the ion and water flow through the pore was considered as a random walk on the reaction diagram. This generates a random queue of ion and water molecules, and the Maxwell demon splits them into a piece of sequence and assigns them to one-way cyclic paths (Fig. 1). Accordingly, one realizes the permeation process as transitions among cyclic paths rather than transitions among states. Each cycle, driven by the conjugated driving force, produces the cycle flux that can be calculated from the rate constants of DSMM. Thus, the experimental observables, such as the net flux, the net ionic current, and the *CR*_w-i_, can be calculated as weighted sums of the cycle fluxes through taking into account the stoichiometric numbers assigned for each cycle. The cyclic path is an elementary unit in the mesoscopic level of permeation hierarchy, and transitions among cyclic paths serve more integrated picture of permeation processes.

Recently, systematic approaches to constructing DSMM from the microscopic trajectories of computer simulation have been developed [34], [35]. Ensembles of microscopic trajectories were collected and lumped, and “states” having transition paths to other states with Markovian processes have been defined. Although this bottom-up approach has not reportedly been applied to ion permeation issues, DSMM can now serve as a connecting point for microscopic trajectories and macroscopic observables.

Hill's expanded diagram is a systematic way of searching paths in the diagram, and all the one-way cyclic paths were traced successfully (Fig. 4). Finding paths through decomposing the states in the original diagram into the path-dependent sub-states is the essence of the idea. Assigning the stoichiometric number as attributes of cyclic paths gave several benefits on the cycle flux analysis. (1) Non-zero cycles and zero cycles were distinguished on DSMM. (2) Accordingly, a mesoscopic structure emerged on DSMM. Some of the cyclic paths are important and others are not. (3) Not only the net flux, but other macroscopic observables, such as the ionic current and the *CR*_w-i_ values were readily calculated, otherwise have been estimated by the Monte Carlo simulation.

In earlier studies, applied DSMM for various channels has been simpler relative to the KcsA model, and the issues raised in the present study have not been recognized. For example, the net flux for simple diagrams was calculated from the transition flux. In the case of the gA model, this is valid. However, it has not been obvious as to whether this simple strategy is applicable when DSMM becomes complicated. Finally, we proved for the first time that the traditional method is not valid for the KcsA model, and the cycle flux algebra gave the exact solution. Furthermore, the calculation of the cycle flux readily predicted the significant changes in the *CR*_w-i_ values at different K^+^ concentrations.

In this study we demonstrated a mesoscopic view point for analyzing permeation processes [31]. Experimental observables were related to the rate constants through the cycle flux algebra. Furthermore, macroscopic observables are decomposed into linear combinations of the cycle fluxes. The cycle kinetics-based approach presented here opens a potential niche in the field of permeation studies.

## Supporting Information

Appendix S1 (DOC)
