# Supplementary material for: Cycle Flux Algebra for Ion and Water Flux through the KcsA Channel Single-File Pore Links Microscopic Trajectories and Macroscopic Observables
Source: PLoS One. 2011 Jan 31;6(1):e16578. doi: 10.1371/journal.pone.0016578 (PMC3031593; doi:10.1371/journal.pone.0016578)
Supplement: Appendix S1 — (DOC) [file pone.0016578.s001.doc]

# **Supporting Information-TEXT S1**

**Cycle Flux Algebra for Ion and Water Flux through the KcsA Channel Single-file Pore Links Microscopic Trajectories and Macroscopic Observables**

Shigetoshi Oiki, Masayuki Iwamoto and Takashi Sumikama

Department of Molecular Physiology and Biophysics,

University of Fukui Faculty of Medical Sciences, Yoshida, Fukui, Japan.

## ***The net flux of the gramicidin A channel calculated from the original and expanded diagrams***

From the expanded diagram (Fig. 2C) the steady-state probability of the sub-states (P_i_) were calculated,

$P_{1a}=k_{31}k_{24}k_{43}/\Sigma$;

$P_{1b}=k_{23}k_{31}\left(k_{43}+k_{42}\right)/\Sigma$;

$P_{1c}=k_{21}\left\{k_{34}k_{42}+k_{32}\left(k_{43}+k_{42}\right)+k_{31}\left(k_{43}+k_{42}\right)\right\}/\Sigma$;

$P_{2}=\left[k_{23}k_{34}k_{42}+k_{32}\left(k_{13}+k_{12}\right)\left(k_{43}+k_{42}\right)+k_{12}\left\{k_{34}k_{42}+k_{31}\left(k_{43}+k_{42}\right)\right\}\right]/\Sigma$;

$P_{3a}=k_{24}k_{43}\left(k_{13}+k_{12}\right)/\Sigma$;

$P_{3b}=k_{23}\left(k_{13}+k_{12}\right)\left(k_{43}+k_{42}\right)/\Sigma$;

$P_{3c}=k_{13}k_{21}\left(k_{43}+k_{42}\right)/\Sigma$;

$P_{4b}=k_{23}k_{34}\left(k_{13}+k_{12}\right)/\Sigma$;

$P_{4c}=k_{13}k_{21}k_{34}/\Sigma$ (Eq. S1)

where

$\begin{matrix} \Sigma=k_{23}k_{31}k_{42}+k_{32}k_{21}k_{43}+k_{32}k_{21}k_{42}+k_{23}k_{31}k_{43} \\ +k_{21}k_{34}k_{42}+k_{31}k_{24}k_{43}+k_{21}k_{31}k_{42}+k_{21}k_{31}k_{43} \\ +k_{32}k_{13}k_{42}+k_{32}k_{13}k_{42}+k_{32}k_{12}k_{42}+k_{32}k_{13}k_{43} \\ +k_{12}k_{34}k_{42}+k_{13}k_{34}k_{42}+k_{12}k_{31}k_{42}+k_{12}k_{31}k_{43} \\ +k_{23}k_{13}k_{42}+k_{23}k_{12}k_{43}+k_{23}k_{12}k_{42}+k_{23}k_{13}k_{43} \\ +k_{12}k_{24}k_{43}+k_{13}k_{24}k_{43}+k_{13}k_{21}k_{42}+k_{13}k_{21}k_{43} \\ +k_{32}k_{13}k_{24}+k_{23}k_{12}k_{34}+k_{32}k_{12}k_{24}+k_{23}k_{13}k_{34} \\ +k_{12}k_{24}k_{34}+k_{13}k_{24}k_{34}+k_{12}k_{31}k_{24}+k_{13}k_{21}k_{34} \end{matrix}$ (Eq. S2)

From these probabilities of substates the one-way cycle flux was obtained.

$J_{a+}=\frac{k_{32}k_{13}k_{21}\left(k_{43}+k_{42}\right)}{\Sigma}$, $J_{a-}=\frac{k_{23}k_{12}k_{31}\left(k_{43}+k_{42}\right)}{\Sigma}$, $J_{b+}=\frac{k_{32}k_{24}k_{43}\left(k_{13}+k_{12}\right)}{\Sigma}$, $J_{b-}=\frac{k_{23}k_{34}k_{42}\left(k_{13}+k_{12}\right)}{\Sigma}$ (Eq. S3)

The net flux (*J*_net_) is expressed as Eq. 7 in the text.

In parallel, the net flux was calculated from the original diagram. The steady-state probability of the states (*p*_i_) was calculated from inversion of the 4 × 4 matrix. *J*_net_ is given as,

$J_{net}=k_{32}p_{3}-k_{23}p_{2}$ (Eq. S4)

where

$$p_{3}=\frac{k_{23}k_{13}k_{43}+k_{23}k_{12}k_{43}+k_{13}k_{21}k_{43}+k_{13}k_{24}k_{43}+k_{12}k_{24}k_{43}+k_{23}k_{13}k_{42}+k_{23}k_{12}k_{42}+k_{13}k_{21}k_{42}}{\Sigma}$$

$p_{2}=\frac{k_{32}k_{13}k_{43}+k_{32}k_{12}k_{43}+k_{12}k_{31}k_{43}+k_{32}k_{13}k_{42}+k_{32}k_{12}k_{42}+k_{12}k_{31}k_{42}+k_{13}k_{34}k_{42}+k_{12}k_{34}k_{42}}{\Sigma}$ (Eq. S5)

Thus, *J*_net_ from the original diagram and that from the expanded diagram was identical.

## ***The matrix for the original diagram of KcsA channel***

For the calculation of the steady-state probability of the states, the transition matrix was formulated from the original diagram

$\left[\begin{matrix} -\left(k_{1}+k_{4}+k_{5}\right) & k_{1}+k_{4} & k_{5} & 0 & 0 & 0 & 0 \\ k_{2}+k_{3} & -\left(k_{2}+k_{3}+k_{6}\right) & 0 & k_{6} & 0 & 0 & 0 \\ k_{7} & 0 & -\left(k_{7}+k_{10}+k_{11}+k_{15}\right) & k_{10} & k_{11} & k_{15} & 0 \\ 0 & k_{8} & k_{9} & -\left(k_{8}+k_{9}+k_{12}+k_{16}\right) & k_{12} & 0 & k_{16} \\ 0 & 0 & k_{13} & k_{14} & -\left(k_{13}+k_{14}+k_{19}+k_{20}\right) & k_{20} & k_{19} \\ 0 & 0 & k_{17} & 0 & k_{22} & -\left(k_{17}+k_{22}\right) & 0 \\ 0 & 0 & 0 & k_{18} & k_{21} & 0 & -\left(k_{18}+k_{21}\right) \end{matrix}\right]$(Eq. S6)

The steady-state probabilities of the states were numerically calculated.

## ***Computability of the net flux from the original diagram***

The net flux can be calculated from the original diagram through the transition flux, if the diagram is simple, like that of the gA channel. On the other hand, for the KcsA channel, combinations of the transition fluxes (e.g., Eq. 12, 13) are not likely to attain the net flux (Eq. 11). Here, we examine the issue whether the net flux could be calculated as a linear combination of the transition fluxes. If it is the case, the cycle flux calculation is not necessary, but if not, the cycle flux calculation through drawing the expanded diagram is mandatory.

Here existence or absence of the solution for the net flux from the transition fluxes was examined algebraically. First, *J*_ij_s were expressed with the cycle fluxes, *J*_κ_s. For the gA channel, there are 5 transition fluxes (rows; *J*_24_, *J*_43_, *J*_32_, *J*_21_, and *J*_13_) and 2 cycle fluxes (columns; *J*_a-b_; *J*_c_ is a zero-cycle), and the following matrix for the relationship between the cycle fluxes and the transition fluxes (*J*_ij_ = A *J*_κ_) was attained.

$A=\left[\begin{matrix} 0 & 1 \\ 0 & 1 \\ 1 & 1 \\ 1 & 0 \\ 1 & 0 \end{matrix}\right]$ (Eq. S7)

For example, the first row represents *J*_24_, which is composed of *J*_b_.

*J*_net_ is simply the sum of *J*_κ_s (Eq. 11). Thus, if any linear combinations of *J*_ij_s would contain all the *J*_κ_s, then *J*_net_ could be computable from *J*_ij_s. For this simple case the solution is trivial. Existence or absence of solution can be proven through formalizing the relationships between *J*_ij_ and *J*_κ_ with a system of linear equation as follows.

$A^{T}\bullet\left[\begin{matrix} w_{1} \\ w_{2} \\ w_{3} \\ w_{4} \\ w_{5} \end{matrix}\right]=\left[\begin{matrix} 1 \\ 1 \end{matrix}\right]$ (Eq. S8)

*T* represents the transpose of the matrix and *w*_i_ the weight factor. Existence of the solution is proven when the ranks of matrix A and the following matrix (adding the solution vector to the matrix A; matrix B) are the same [1].

$B=\left[\begin{matrix} 0 & 1 \\ 0 & 1 \\ 1 & 1 \\ 1 & 0 \\ 1 & 0 \\ 1 & 1 \end{matrix}\right]$ (Eq. S9)

The rank of matrix A was 2 and that for matrix B was 2, and it was proven that *J*_net_ can be computable from a linear combinations of *J*_ij_s.

In the case of the KcsA diagram, there are 11 transition fluxes (rows; *J*_12_, *J*_21_, *J*_13_, *J*_42_, *J*_43_, *J*_35_, *J*_54_, *J*_36_, *J*_74_, *J*_65_, and *J*_57_) and 14 cycle fluxes (columns; *J*_a-n_) and the following matrix is:

$A=\left[\begin{matrix} 1 & 0 & 0 & 0 & 0 & 0 & 0 & 0 & 0 & 0 & -1 & -1 & -1 & -1 \\ 1 & 1 & 1 & 1 & 1 & 0 & 0 & 0 & 0 & 1 & 0 & 0 & 0 & 0 \\ 0 & 1 & 1 & 1 & 1 & 0 & 0 & 0 & 0 & 1 & 1 & 1 & 1 & 1 \\ 0 & 1 & 1 & 1 & 1 & 0 & 0 & 0 & 0 & 1 & 1 & 1 & 1 & 1 \\ 0 & 0 & 0 & 0 & 0 & 1 & 1 & 1 & 1 & -1 & 0 & 0 & 0 & 0 \\ 0 & 1 & 0 & 0 & 1 & 1 & 0 & 0 & 1 & 0 & 1 & 0 & 1 & 0 \\ 0 & 1 & 0 & 1 & 0 & 1 & 0 & 1 & 0 & 0 & 1 & 1 & 0 & 0 \\ 0 & 0 & 1 & 1 & 0 & 0 & 1 & 1 & 0 & 0 & 0 & 1 & 0 & 1 \\ 0 & 0 & 1 & 0 & 1 & 0 & 1 & 0 & 1 & 0 & 0 & 0 & 1 & 1 \\ 0 & 0 & 1 & 1 & 0 & 0 & 1 & 1 & 0 & 0 & 0 & 1 & 0 & 1 \\ 0 & 0 & 1 & 0 & 1 & 0 & 1 & 0 & 1 & 0 & 0 & 0 & 1 & 1 \end{matrix}\right]$ (Eq. S10)

For example, the fifth row represents *J*_43_, which is composed of *J*_f_, *J*_g_, *J*_h_, *J*_i_, and -*J*_j_. A system of linear equation is as follows.

$A^{T}\bullet\left[\begin{matrix} w_{1} \\ w_{2} \\ w_{e} \\ w_{4} \\ w_{5} \\ w_{6} \\ w_{7} \\ w_{8} \\ w_{9} \\ w_{10} \\ w_{11} \end{matrix}\right]=\left[\begin{matrix} 1 \\ 1 \\ 1 \\ 1 \\ 1 \\ 1 \\ 1 \\ 1 \\ 1 \\ 1 \\ 1 \\ 1 \\ 1 \\ 1 \end{matrix}\right]$ (Eq. S11)

while matrix B is

$B=\left[\begin{matrix} 1 & 0 & 0 & 0 & 0 & 0 & 0 & 0 & 0 & 0 & -1 & -1 & -1 & -1 \\ 1 & 1 & 1 & 1 & 1 & 0 & 0 & 0 & 0 & 1 & 0 & 0 & 0 & 0 \\ 0 & 0 & 0 & 0 & 0 & 1 & 1 & 1 & 1 & -1 & 0 & 0 & 0 & 0 \\ 0 & 1 & 1 & 1 & 1 & 0 & 0 & 0 & 0 & 1 & 1 & 1 & 1 & 1 \\ 0 & 1 & 1 & 1 & 1 & 0 & 0 & 0 & 0 & 1 & 1 & 1 & 1 & 1 \\ 0 & 1 & 0 & 0 & 1 & 1 & 0 & 0 & 1 & 0 & 1 & 0 & 1 & 0 \\ 0 & 1 & 0 & 1 & 0 & 1 & 0 & 1 & 0 & 0 & 1 & 1 & 0 & 0 \\ 0 & 0 & 1 & 1 & 0 & 0 & 1 & 1 & 0 & 0 & 0 & 1 & 0 & 1 \\ 0 & 0 & 1 & 0 & 1 & 0 & 1 & 0 & 1 & 0 & 0 & 0 & 1 & 1 \\ 0 & 0 & 1 & 1 & 0 & 0 & 1 & 1 & 0 & 0 & 0 & 1 & 0 & 1 \\ 0 & 0 & 1 & 0 & 1 & 0 & 1 & 0 & 1 & 0 & 0 & 0 & 1 & 1 \\ 1 & 1 & 1 & 1 & 1 & 1 & 1 & 1 & 1 & 1 & 1 & 1 & 1 & 1 \end{matrix}\right]$ (Eq. S12)

The rank of matrix A was 5 and that for matrix B was 6, and it was proven that no linear combinations of *J*_ij_s give *J*_net_.

**References**

1. Strang G (2004) Linear Algebra and Its Applications. Pacific Grove: Brooks Cole.
